# How to Protect Ovarian Function before and during Chemotherapy?

**DOI:** 10.3390/jcm10184192

**Published:** 2021-09-16

**Authors:** Luca Arecco, Tommaso Ruelle, Valentino Martelli, Andrea Boutros, Maria Maddalena Latocca, Stefano Spinaci, Camilla Marrocco, Claudia Massarotti, Matteo Lambertini

**Affiliations:** 1 U.O. Clinica di Oncologia Medica, IRCCS Ospedale Policlinico San Martino, 16132 Genova, Italy; arecco.luca@gmail.com (L.A.); mariamaddalena.latocca@gmail.com (M.M.L.); camilla.marrocco@hotmail.com (C.M.); 2 Department of Internal Medicine and Medical Sciences (DiMI), School of Medicine, University of Genova, 16132 Genova, Italy; tommirue@gmail.com (T.R.); martellivalentino91@gmail.com (V.M.); boutros.andrea@gmail.com (A.B.); 3 U.O. Oncologia Medica 2, IRCCS Ospedale Policlinico San Martino, 16132 Genova, Italy; 4 U.O. Oncologia Medica 1, IRCCS Ospedale Policlinico San Martino, 16132 Genova, Italy; 5 Division of Breast Surgery, Ospedale Villa Scassi ASL3, 16149 Genova, Italy; stefano.spinaci@hotmail.com; 6 Department of Neurosciences, Rehabilitation, Ophthalmology, Genetics, Maternal and Child Health (DiNOGMI), School of Medicine, University of Genova, 16132 Genova, Italy; claudia.massarotti@gmail.com; 7 Academic Unit of Obstetrics and Gynaecology, IRCCS Ospedale Policlinico San Martino, 16132 Genova, Italy

**Keywords:** cancer, premenopausal patients, ovarian function preservation, premature ovarian insufficiency

## Abstract

A significant number of women receive a cancer diagnosis before their age of natural menopause. Among these patients, the most frequent neoplasms are breast cancer, gynecological, and hematological malignancies. Premature ovarian insufficiency and infertility are among the most feared short- to long-term consequences of anticancer treatments in premenopausal patients. Both patient- and treatment-related characteristics are key factors in influencing the risk of gonadotoxicity with the use of chemotherapy. The cryopreservation of oocytes/embryos is a standard strategy for fertility preservations offered to young women interested in future family planning, but it does not allow gonadal function protection during chemotherapy. Ovarian suppression with gonadotropin-releasing hormone agonist (GnRHa) during chemotherapy is now recommended as an option to reduce the risk of gonadotoxicity in order to avoid the negative consequences of premature ovarian insufficiency in premenopausal women receiving cytotoxic therapy, including those not interested in fertility preservation. This review summarizes the risk of treatment-induced gonadotoxicity in premenopausal patients and the evidence available on the protective role of administering GnRHa during chemotherapy to preserve ovarian function.

## 1. Introduction

Thanks to improvements in cancer prognosis, increased attention should be paid to the future quality of life for patients exposed to anticancer treatments [1]. The risk of treatment-induced gonadotoxicity is among the most feared short- and long-term time side effects of anticancer therapies in premenopausal women [2]. Developing premature ovarian insufficiency (POI) and infertility are of high concern to many patients [3]. Therefore, trying to reduce the impact of these side effects is of primary importance [4]. The most frequent neoplasms diagnosed in premenopausal women and associated with the risk of developing these side effects are breast, gynecological and hematological malignancies [5].

As recommended by current guidelines, proper oncofertility counseling is recommended in all premenopausal women irrespective of the type of their disease and stage [1,6]. During counseling, patients should be informed about the risk of developing POI with the proposed treatment and the available strategies to counteract this side effect. The difference between fertility and ovarian function preservation should be explained, as well as the different eligibility criteria needed to access the available strategies.

The cryopreservation of oocytes/embryos and/or ovarian tissue are standard strategies for fertility preservation in young women who are interested in future family planning [1,6]. These strategies should be offered preferably to young women with a suggested age cut-off of 40 and 36 years for oocyte/embryo and ovarian tissue cryopreservation, respectively [1]. While ovarian function recovery is possible after ovarian tissue cryopreservation, cryopreserving oocytes/embryos does not protect against the risk of treatment-induced POI. For ovarian function preservation, the only recommended medical treatment is the administration of gonadotropin-releasing hormone agonist (GnRHa) during chemotherapy [1,6]. This strategy has been developed as an option to reduce the risk of gonadotoxicity in order to avoid the negative consequences of POI in premenopausal women (including patients older than 40 years at diagnosis) receiving cytotoxic therapy [7]. Hence, this is a strategy that can be offered also to patients who are not interested in fertility preservation.

As recently shown in the prospective PREFER study, while less than 20% of young women diagnosed with breast cancer before the age of 40 years decided to undergo cryopreservation strategies for fertility preservation, more than 90% of them accepted the use of GnRHa during chemotherapy [8]. Moreover, 90.6% of patients aged 41–45 years at diagnosis (i.e., those normally not candidates to fertility preservation strategies) decided to receive GnRHa during chemotherapy to reduce the risk of POI and its consequences [8]. The impact of estrogen deficiency due to an early loss of ovarian function on the quality of life and general health of these patients in the long-term should not be undervalued [9]. Therefore, it is important for clinicians involved in cancer care to properly discuss POI risk as well as the importance of ovarian function preservation.

This review summarizes the risk of treatment-induced gonadotoxicity in premenopausal patients and the available evidence on the protective role of administering GnRHa during chemotherapy to preserve ovarian function.

## 2. Risk of Chemotherapy-Induced Ovarian Damage

### 2.1. Cytotoxic Agents

To estimate the risk of treatment-induced gonadotoxicity, both patient- and treatment-related factors should be considered during counseling [1,6]. Regarding the patient, the most important factor to influence this risk is represented by the age at the time of treatment: the same treatment is associated with higher POI risk the older the age of the patient (i.e., the closer the woman is to her natural age at menopause) [1,6]. Genetic factors may also play a role. Germline pathogenic variants in BRCA genes have shown to be potentially associated with a reduced ovarian reserve at diagnosis [10]. However, limited and conflicting data are available on their role in influencing the risk of treatment-induced gonadotoxicity [11,12,13].

In terms of treatment-related factors, the type and dose of chemotherapy have a major impact on the risk of gonadotoxicity [1,6].

In general, different mechanisms are associated with the ovarian toxicity of chemotherapy. The germ cells pool can be damaged through a direct effect on double-strand DNA or acceleration in follicular activation [14], but also with an indirect impact on the stroma in terms of decrease of blood vessels and reduction of blood supply [15].

Alkylating agents, such as cyclophosphamide, are widely used for the treatment of many malignancies. These are the agents associated with the highest gonadotoxic impact [16]. A metanalysis by Zhao et al. demonstrated that cyclophosphamide-based regimens can lead to a higher incidence of amenorrhea compared to the cyclophosphamide-free ones (OR 2.25; 95% CI 1.26–4.03, *p* = 0.006) [17]. The impact of different chemotherapy regimens, also according to the patients’ age at the time of treatment, has been recently summarized in international guidelines [1,6].

### 2.2. Monoclonal Antibodies

Monoclonal antibodies are now routinely administered in clinical practice in different malignancies. Doubts remain about their actual gonadotoxicity, as they are often combined with other chemotherapeutic drugs. Limited data exist on the possible effects on ovarian function and fertility of anti-HER2 therapies.

The ALTTO trial suggested the gonadal safety of trastuzumab and/or lapatinib [18]. The rate of amenorrhea was 72.6% with trastuzumab alone, 74.0% with lapatinib alone, and 74.8% and 72.1% with combination and sequential use, respectively (*p* = 0.64). However, all patients received prior chemotherapy and there was no control arm without anti-HER2 agents [18].

Other data about the gonadotoxicity of trastuzumab derive from the APT trial. Ruddy et al. evaluated the incidence of menstrual impairment in the premenopausal subgroup population receiving weekly paclitaxel with trastuzumab for 3 months, followed by trastuzumab monotherapy for completing one year of anti-HER2 therapy [18]. A lower rate of premature ovarian insufficiency (28%) was shown with this treatment as compared to what is expected with other breast cancer adjuvant chemotherapy regimens [18].

### 2.3. PARP Inhibitors

The poly (ADP-ribose) polymerase (PARP) inhibitors are administered in patients harboring germline pathogenic variants in *BRCA1/2*. They are frequently employed in patients affected by breast or gynecological cancers, which account for a large proportion of premenopausal patients. There is some evidence about their effects on the gonads, even if extracted from mouse models [19]. Winship et al. assessed the activity of cyclophosphamide, doxorubicin and other anticancer drugs alone, or in combination with olaparib in *BRCA* wild-type murine ovarian cells. The primordial follicles have been found as the most affected by the combined regimen and in particular it has been observed the depletion of more than a third of their pool (*p* < 0.05) [19]. These agents may be soon available in the early setting; hence, it is imperative to highlights the need to investigate their gonadotoxicity in the clinical scenario.

## 3. De-Escalation of Gonadotoxic Chemotherapy

In recent years, alongside with increased therapeutic options in the treatment of early-stage cancer, a growing attention has been paid to tailoring the type and intensity of systemic therapies, while balancing between the individual risk of relapse and the toxicity caused by these therapies [20,21,22,23]. This concept of de-escalation of treatment applies also to finding alternative regimens with a reduced gonadotoxicity in premenopausal patients.

Breast cancer represents a clear example. The approach of reducing the number and the length of anticancer therapies has been investigated in depth in patients with HER2-positive breast cancer, thanks to the growing availability of very effective anti-HER2 agents [20,21,22]. As an example, the APT trial is a study that recruited 406 patients with node-negative pT < 3 cm HER2-positive breast cancer who underwent upfront surgery [21]. These patients received an anthracycline- and cyclophosphamide-free adjuvant regimen with weekly paclitaxel and trastuzumab followed by trastuzumab alone. After a median follow-up of 6.5 years, invasive disease-free survival (iDFS) rate was 93% with a 7-years overall survival (OS) of 95%, demonstrating the excellent prognosis of these patients, even when omitting anthracycline and cyclophosphamide from standard chemotherapy [21]. This is highly relevant for avoiding the toxicities of the anthracycline and cyclophosphamide chemotherapy component, which include the risk of gonadotoxicity in premenopausal women. In this trial, data on the chemotherapy-induced menorrhea were reported [24]. In this analysis, menstrual resumption after cytotoxic treatment was assessed with surveys sent every 6 months to patients during the first 3 years, and then every year for the whole duration of follow-up. Out the 406 patients recruited in the APT trial, 64 were eligible for this analysis being younger than 55 years, at least reporting one menstrual episode in the previous 6 months with a frequency of at least every 2 months (i.e., pre-menopausal status at baseline). Out of the 64 included patients, 18 (28%) were amenorrheic and 46 (72%) had not experienced amenorrhea at the time of the last menstrual survey, with a significant lower rate of chemotherapy-induced amenorrhea as compared to what was expected with standard adjuvant cytotoxic breast cancer regimens that also include an anthracycline and cyclophosphamide. As expected, the median age at study entry of patients who experienced chemotherapy-induced amenorrhea was higher than in patients with menstrual resumption (42 vs. 49 years), reinforcing the evidence that older age is a major risk factor for developing chemotherapy-induced amenorrhea [24].

In the same setting of HER2-positive early breast cancer, another attempt to de-escalate adjuvant chemotherapy in stage I disease is the ATEMPT trial [22]. This study evaluated the efficacy and safety of a one-year of treatment with single-agent trastuzumab emtansine (T-DM1), an antibody-drug conjugate [25], as compared to paclitaxel and trastuzumab [22]. A total of 497 patients were randomly assigned to T-DM1 or paclitaxel with trastuzumab. Out of the 383 patients randomized to T-DM1, 3-year iDFS was 97.8%, with similar results as those observed in the APT trial (98.7%) [22]. Notably, although the percentage of patients who experienced clinically relevant toxicities was almost equal (46% T-DM1 vs. 47% paclitaxel and trastuzumab, *p* = 0.83), a higher rate of toxicity-related treatment discontinuation was observed in patients in the T-DM1 arm (17% vs. 6%). Patient-reported outcomes indicated that patients treated with T-DM1 had a better quality of life compared to those treated with paclitaxel and trastuzumab. The authors investigated the rate of amenorrhea among premenopausal patients recruited in the ATEMPT trial. In this analysis, 18-month chemotherapy-induced amenorrhea (defined as the absence of menses resumption in the previous 6 months) after therapy completion was the primary endpoint [26]. Out of the 383 patients randomized, 123 were premenopausal but only 76 patients (18 in the paclitaxel and trastuzumab arm and 58 in the T-DM1 arm) had menopausal data available at 18 months. The rate of chemotherapy-induced amenorrhea was 50% in the trastuzumab and paclitaxel group and 24% in the T-DM1 group (*p* = 0.045). These rates seemed to increase in the T-DM1 arm after 18 months compared to paclitaxel and trastuzumab, perhaps because a longer duration of T-DM1 administration (12 months versus 3 months of paclitaxel and trastuzumab) can cause a delayed chemotherapy-induced amenorrhea. Although there is a trend towards a reduction in gonadotoxicity compared to standard chemotherapy, future studies are warranted to fully investigate the gonadotoxicity of T-DM1.

An additional example in the HER2-positive setting, but in patients at higher risk of disease recurrence and receiving neoadjuvant treatment, is represented by the TRAIN-2 study that investigated the possibility to reduce the chemotherapy burden by implementing the anti-HER2 blockade [20]. The TRAIN-2 study is a randomized phase III trial in which 438 patients with stage II/III were randomized to receive dual HER2 blockade with pertuzumab and trastuzumab in association with carboplatin and paclitaxel or 5-fluorouracil, epirubicin, and cyclophosphamide, followed by paclitaxel and carboplatin. An equivalent percentage of pathological complete responses (pCR) was observed in both groups (68% in the anthracycline-free group vs. 67% in the anthracycline group), with a significant reduction in serious adverse events (SAE) in the anthracycline-free group (22% vs. 28%). From the recent three-year follow-up analysis, event-free survival (EFS) estimates were 92.7% in the anthracycline group and 93.6% in the anthracycline-free group, while 3-year OS were 97.7% in the anthracycline group and 98.2% in the anthracycline-free group, showing a similar estimated EFS and OS associated with decreased risk of febrile neutropenia, cardiotoxic effects, and secondary malignant neoplasms in the anthracycline-free group [27]. 

Sparing the gonadotoxicity of anthracyclines and cyclophosphamide in premenopausal women would be of great importance and is the standard of care in low risk HER2-positive breast cancer [28], but further studies are needed to refine the selection of patients with other tumor subtypes or with HER2-positive disease but at higher risk of recurrence that can be candidates to such de-escalated approach.

A recent study in women with HER2-negative/estrogen receptor-positive early breast cancer investigated a de-escalated chemotherapy approach with the main aim of reducing its gonadotoxicity by removing the cyclophosphamide component without compromising its prognosis. This phase III trial randomized 521 breast cancer patients aged <40 after upfront surgery to receive standard chemotherapy with epirubicin and cyclophosphamide, followed by weekly paclitaxel (EC-wP) or a cyclophosphamide-free regimen (epirubicin and paclitaxel, which was then followed by weekly paclitaxel [EP-wP]) [23]. The co-primary endpoints were 5-years iDFS and rate of menstrual resumption at 12 months after chemotherapy, defined as two consecutive menstruations, or only one menstruation with pre-menopausal levels of estradiol and follicle-stimulating hormone. After a median follow-up of 62 months, 5-years iDFS was 84.7% in the EP-wP arm (95% CI 79.3% to 88.8%) and 78.3% in the EC-wP arm (95% CI 72.2% to 83.3%). When considering the other co-primary endpoint, a significantly higher rate of menstrual resumption was observed in patients treated without cyclophosphamide (63.1% in EP-wP vs. 48.3% in EC-wP, 95% CI 42.2% to 54.3%) with an absolute difference of 14.8% (95% CI 6.37% to 23.2%, *p* < 0.001). In the post hoc exploratory analysis of pregnancy outcomes, of the 228 patients analyzed, successful pregnancy occurred in 2.7% women in the EC-wP group and 9.6% in the EP-wP group (*p* = 0.03) [23].

## 4. Ovarian Suppression with GnRHa during Chemotherapy

In order to prevent chemotherapy-induced POI and early menopause-related symptoms, the administration of GnRHa during chemotherapy is to date the only medical strategy available for clinical use [1,6,29]. To date, the mechanisms underlying the protective role of ovarian suppression with GnRHa during chemotherapy have not been fully understood [30]; this is the main reason for the long debate around the role of this strategy [31]. However, several randomized trials have clarified its efficacy and safety, particularly in premenopausal women with early breast cancer.

### 4.1. Mechanisms of Action

After the pubertal age, most of the follicles are quiescent at the primordial stage; after reaching prenatal stage, follicle development depends on the secretion of gonadotropins (follicles-stimulating hormone [FSH] and luteinizing hormone [LH]), regulated by the secretion of GnRH by the hypothalamus [7]. Chemotherapy-induced gonadotoxicity involves all follicular stages and cell types, impairing both ovarian reserve and hormonal function through direct and indirect damages [32]. To date, the mechanism underlying the protective gonadal effect of GnRHa is not fully clear, but this strategy seems to have both indirect and direct effects on the ovaries.

#### 4.1.1. Indirect Effects

The protective action of GnRHa was initially attributed to the reaching of a pre-pubertal hormonal state [7]. The administration of GnRHa induces an initial release of gonadotropin, which causes a desensitization of GnRH-receptors and prevents from the effects of pulsatile GnRH secretion (the “flare-up effect”) [33]. This condition would be able to generate a hypogonadotropic state that keeps the follicles in a quiescent state, making them less vulnerable to chemo-induced damage [34]. Cytotoxic agents cause the apoptosis of follicles, decreasing the levels of estrogens and inhibin and, accordingly, increased the levels of FSH, which stimulates the recruitment of primordial follicles (the so called “burn-out effect of chemotherapy”). Therefore, the GnRH-induced FSH suppression slows down the proliferation of follicular cells, protecting them from the cytotoxic damage and delaying the recruitment of the follicular pool of quiescent cells [33].

In addition, proliferating follicles also release anti-müllerian hormone (AMH), which can negatively regulate the primordial follicles recruiting. During chemotherapy, AMH levels are usually dramatically lowered, causing a recruitment of primordial follicles and exposing them to chemo-induced damage. It has been observed that the addition of GnRHa can raise AMH levels and prevent this effect [35,36].

Finally, despite a limited number of data, another potential indirect protective mechanism of GnRHa is the reduction of utero-ovarian perfusion, which would also reduce the exposure of follicles to the gonadotoxic effect of treatments [29].

#### 4.1.2. Direct Effects

It has been observed that GnRH receptors are expressed on the surface of the ovarian cells and that their activation may result in an anti-apoptotic effect; this effect is currently poorly understood [37,38].

Furthermore, GnRHa may be involved in the upregulation of the anti-apoptotic molecule sphingosine-1-phosphate (S1P), which inhibits the ceramide pathway involved in chemo-induced apoptosis of the ovarian cells. Moreover, S1P improves neo-angiogenesis in primordial ovarian follicles, producing a potential further protective effect in the ovaries [39]. It has been observed that S1P has an anti-apoptotic effect on oocytes exposed in vivo and in vitro to chemotherapy [40,41], but there is still no experimental evidence that GnRHa administration increases level of S1P or other anti-apoptotic molecules. It has also been found that germ line stem cells are present in the ovarian tissue, which are able to reconstitute the primordial follicle pool [42]. GnRHa administration may promote the activation of primordial follicles after the completion of chemotherapy, interacting with these germ cells [43,44].

Recently, Scaruffi et al. evaluated the effect of chemotherapy (i.e., cyclophosphamide) and GnRHa alone, or in combination with chemotherapy on a culture system of ex vivo human immature cumulus cell-oocyte complexes. The effects of these treatments were evaluated on GnRH receptors, ceramide, and apoptosis pathways and glutathione synthesis. This study demonstrated that cyclophosphamide was mainly detrimental to the cumulus cells compartment through the activation of apoptosis molecular signals. Furthermore, it was found that GnRHa co-administration downregulated the expression of some pro-apoptotic genes and up-regulated transcription of the anti-apoptotic gene only in cumulus cells with an indirect protective effect on oocytes [45]. These results are in contrast with previous data that excluded a protective effect of GnRHa against cyclophosphamide in ex vivo and in vitro models of human ovary and granulosa cells [46]. The authors suggested that this difference may be due both to the different timing of drug administration and to the different dose of GnRHa used for experiments. In fact, in the work by Scaruffi et al. GnRHs was administered earlier before the addition of chemotherapy and with a higher dose than in the model of Bildik and colleagues.

### 4.2. Clinical Data in Breast Cancer

Most of the evidence supporting the use of GnRHa in the prevention of chemotherapy-induced POI exists in premenopausal women with early breast cancer [47]. A total of 15 randomized trials have been conducted in this setting (Table 1) [48,49,50,51,52,53,54,55,56,57,58,59,60,61,62,63,64]. 

In this regard, most studies have reported results at such a short follow-up that an appropriate assessment of fertility preservation potential was not possible, especially in patients who are often candidates for adjuvant endocrine therapy for 5 to 10 years [66,67,68]. Moreover, the majority of the randomized studies also included premenopausal patients older than 40 years; most notably, the desire for conception was not an inclusion criteria. It should be highlighted that only the POEMS/SWOG S0230 study considered the number of post-treatment pregnancies as a pre-planned endpoint [60,63]. The study showed that the use of temporary ovarian suppression with GnRHa during chemotherapy was associated with a higher number of post-treatment pregnancies [60,63]. GnRHa administration was associated with a significantly higher number of patients conceiving after treatments also in the updated analysis of the PROMISE-GIM6 trial [58] and the Anglo Celtic Group OPTION study [61], but the absolute numbers were small and differences not significant. 

Two safety concerns have been highlighted regarding temporary ovarian suppression with GnRHa during chemotherapy, particularly in patients with estrogen receptor-positive breast cancer: a potential antagonism of endocrine therapy with chemotherapy and a possible negative prognostic effect of POI prevention [69].

However, several randomized clinical trials did not demonstrate any difference in the prognosis of patients who received ovarian suppression during chemotherapy [70,71,72]. The TEXT and SOFT trials have also confirmed this evidence, showing no difference in the survival outcomes of premenopausal women with estrogen receptor-positive breast cancer treated with GnRHa before or following chemotherapy [73].

Furthermore, two randomized trials that evaluated the administration of GnRHa during chemotherapy also in patients with estrogen receptor-positive breast cancer and its impact on survival showed no difference in the survival outcomes; in both studies, most of the patients were also treated with GnRHa as part of their adjuvant endocrine therapy [58,62]. Recently, the results of the final analysis of the PROMISE-GIM6 trial at a median follow-up of 12.4 years have become available. This updated analysis confirmed no difference in 10-year DFS nor in 10-year OS between patients treated with chemotherapy alone, or with concurrent GnRHa, including among the cohort of women with hormone receptor-positive disease [65]. These results are reassuring concerning the safety of GnRHa use during chemotherapy as a strategy for preserving ovarian function in premenopausal patients irrespective of the hormone receptor status of their tumor. 

Nevertheless, some considerations are needed to better interpret the results of these trials; most of the studies had a small sample size including less than 100 patients, median age of the included patients was close to 40 years in most of them, and the most used chemotherapy regimen was an anthracycline- and cyclophosphamide-based regimen. However, it should be remarked that the definition of chemotherapy-induced POI was based only on menstrual function after treatment in the majority of the studies. Only few studies used a composite endpoint that included both amenorrhea and post-menopausal hormonal levels and the timing for POI evaluation was also different, ranging from 6 months up to more than 5 years after the end of chemotherapy treatment. To date, there is no uniform and accepted definition of chemotherapy-induced POI. This is reflected by the use of different definitions that in most cases included amenorrhea, and in the timing of its assessment. However, amenorrhea is not an optimal surrogate marker to define the gonadotoxicity of anticancer treatments, especially in breast cancer patients who often receive adjuvant endocrine therapies (including tamoxifen), which can further impact menstrual function recovery. Experts recommend empirically to define chemotherapy-induced POI with a composite definition of amenorrhea for ≥2 years and a post-menopausal hormonal profile [6,74]. Notably, only few studies reported an assessment of menstrual function at long-term and no trials assessed the final age at menopause. In the studies that evaluated AMH [51,59,61,64,75,76,77,78], no difference in post-treatment AMH levels was observed between patients who received GnRHa during chemotherapy and those treated with systemic cytotoxic therapy alone. However, in all these studies, AMH was assessed in only a small proportion of randomized patients, limiting the interpretation of these results.

Results from the available randomized trials have been summarized in several meta-analyses in order to better define the efficacy and safety of temporary ovarian suppression with GnRHa during chemotherapy in breast cancer patients (Table 2) [79,80,81,82,83,84,85,86,87]. Except for 2 meta-analyses, all the others demonstrated a protective effect of GnRHa administration in reducing the risk of chemotherapy-induced POI with a clearer benefit when only trials conducted in breast cancer were included. The largest and most recent meta-analyses, including the only based on individual patient level data [87], showed also a significantly higher rate of post-treatment pregnancy rate [83,84,86,87,88].

### 4.3. Clinical Data in Patients with Malignancies Other Than Breast Cancer

The use of GnRHa during chemotherapy as a strategy to preserve ovarian function has been considerably less investigated in women with malignancies other than breast cancer (Table 3) [75,76,77,78,89,90].

Four randomized trials have been conducted in premenopausal women with lymphoma [75,76,78,89]. In three of these trials, only premenopausal women with Hodgkin’s lymphoma were included, while both patients with Hodgkin’s and non-Hodgkin’s lymphoma were included in the trial by Demeestere and colleagues. In two of these trials, chemotherapy-induced POI was defined only based on menstrual resumption after treatment, while in the other two trials it was defined on post-menopausal hormone levels after chemotherapy. In none of these trials a composite endpoint of menstrual bleeding and hormone levels was used. The timing of POI assessment was highly variable, ranging from 6 months to more than 5 years after the end of chemotherapy. In addition, it should be specified that all trials included a small number of patients, exceeding 30 patients only in the trial by Demeestere and colleagues. The chemotherapy regimens were highly different in terms of the risk of gonadotoxicity, including low-risk regimens (e.g., ABVD including doxorubicin, bleomycin, vinblastine, and dacarbazine) and high-risk regimens (such as induction regimens in hematopoietic stem cell transplants). With the limits of heterogeneous endpoints and timing of evaluation of the ovarian function, and with limited number of patients, all these four trials did not demonstrate a protective effect of temporary ovarian suppression with GnRHa during chemotherapy.

Only one study on the efficacy of temporary ovarian suppression with GnRHa during chemotherapy was conducted in premenopausal women with ovarian cancer. A total of 30 premenopausal women were randomized to receive chemotherapy with or without GnRHa. All women who received GnRHa during chemotherapy had a resumption of menstrual bleeding at 6 months after the end of their cytotoxic treatments, whereas 33% of women who did not receive GnRHa had chemotherapy-induced POI (*p* = 0.02) [73,90].

There are potential clinical and methodological reasons for the different results observed in patients with hematological malignancies as compared to those in the breast cancer setting.

From a clinical point of view, there are important differences between premenopausal patients with hematological diseases, particularly lymphomas, and those with breast cancer. In general, patients with hematological malignancies are characterized by a younger age at diagnosis and are treated with different chemotherapy regimens ranging from a low- to very high-risk of gonadotoxicity. Younger patients generally have a higher ovarian reserve and acute POI is usually observed only after treatment with high-risk chemotherapy regimens, whereas low- or medium-risk chemotherapy are less likely associated with POI in these patients.. On the contrary, premenopausal breast cancer patients have an average older age at diagnosis and often receive chemotherapy regimens characterized by an intermediate risk of gonadotoxicity. Therefore, the protective effect of GnRHa may become visible only in this situation and not in the case of very low or high gonadotoxicity risk.

It should be also mentioned that lymphoma patients may have a reduced ovarian reserve due to their disease even before starting anticancer therapies [91,92], whereas this has not been described so far in breast cancer patients. This issue may have implications on the differences in the obtained results.

From a methodological point of view, only four small studies with a total of 154 patients affected by lymphoma have been conducted, while to date 15 studies involving 1743 patients are available in the breast cancer setting, four of which randomized more than 200 women. The lack of power in trials concerning malignancies other than breast cancer must be considered as an important reason for the controversial results.

Several meta-analyses were performed to gather stronger data in favor of this technique in different types of cancers, and not only breast cancer (Table 4) [32,59,93,94,95,96,97,98,99,100,101,102,103,104].

Among these meta-analyses, all but one showed that the use of GnRHa during chemotherapy has a protective effect on the risk of POI, while only one of them showed that this technique could increase the probability of spontaneous pregnancy in the short-term after the end of treatments [100]. However, only 3 of these meta-analyses [93,97,105] included exclusively patients with malignancies other than breast cancer (HL and NHL and autoimmune diseases in particular).

Several limitations are present in these meta-analyses due to differences in the included trials. First, as mentioned above, different definitions of POI were used in the study and the limited duration of the follow-up of the trials precluded the ability to determine the long-term impact of GnRHa on the preservation of ovarian function and fertility. In addition, a moderate heterogeneity among the studies is also due to the different chemotherapy regimens administered that have been modified in clinical practice over the years and may have partially affected the outcomes of the trials included in the meta-analyses.

## 5. Conclusions

Despite continuous research efforts in this field, many physicians and patients are still concerned about discussing preservation of ovarian function and fertility for the fear that the proposed strategies may have a negative effect on oncological outcomes [8]. Thanks to constant reassuring data about the safety of conceiving following the completion of anticancer treatments [106], it is increasingly important to conduct a proper oncofertility counselling in all patients in order to increase their possibility of completing their family building plan. Even more importantly, it is essential to ensure that all women can experience a quality of life as equal as possible to that of healthy women from the general population. Hence, protecting ovarian function and avoiding all the side effects associated with POI is of primary importance [1,6].

POI development is potentially a direct effect of gonadotoxic therapies in all premenopausal cancer patients; the use of adjuvant endocrine treatments for many years after diagnosis can further amplify this issue [107]. To date, with the exception of de-escalation of chemotherapy in selected patients, the only recognized and approved method to protect ovarian function in premenopausal women undergoing cytotoxic therapy is the concomitant use of GnRHa [1].

However, it must be highlighted that most of the available randomized trials assessing the use of GnRHa during chemotherapy have been conducted in breast cancer patients, with limited and mostly negative evidence in women with hematological malignancies. Nevertheless, also in the setting of hematological malignancies, GnRHa during chemotherapy could be taken into consideration to prevent menometrorrhagia by controlling the menstrual cycle [1]. The main concepts presented in this work are summarized in Table 5.

Importantly, it should be remembered that, for patients interested in fertility preservation, temporary ovarian suppression with a GnRHa during chemotherapy is not an alternative to cryopreservation techniques [1,6].

The main limitation of the current evidence, in addition to the limited data outside the breast cancer field, is the short follow-up of most of the randomized trials without the possibility of evaluating the long-term outcomes, including post-treatment pregnancies and age at menopause. Moreover, collecting information on ovarian reserve markers during treatment and follow-up should be planned in all new trials that want to investigate the effect of cancer treatments on ovarian reserve and the impact on POI.

Besides GnRHa during chemotherapy, other pharmacological and biomedical techniques that can prevent POI in these patients are under constant development [9], but they are still in early phase of development.

## Figures and Tables

**Table 1:** Randomized trials evaluating temporary ovarian suppression with GnRHa during chemotherapy in breast cancer patients.

Authors	Year	POI Definition (Timing of Its Evaluation)	Timing POI Evaluation (Months)	Treatment Regimen	No. Patients	Median Age (Years)	Overall Results
Li et al. [48]	2008	Amenorrhea	12	CT + goserelin CT alone	31 32	40 39	Protection
Badawy et al. [49]	2009	Amenorrhea without resumption of ovulation	8	CT + goserelin CT alone	39 39	30 29.2	Protection
Sverrisdottir et al. [50]	2009	Amenorrhea	Up to 36	CT + goserelin (± tamoxifen) CT (± tamoxifen)	51 43	45 45–46	Protection
Gerber et al. [51]	2011	Amenorrhea	6	CT + goserelin CT alone	30 30	35 38.5	No protection
Sun et al. [52]	2011	Amenorrhea	12	CT + goserelin CT alone	11 10	38 37	Protection
Del Mastro et al. [53] Lambertini et al. [58] Lambertini et al. [65]	2011 2015 2021	Amenorrhea and postmenopausal levels of FSH and E2	12	CT + triptorelin CT alone	148 133	39 39	Protection
Munster et al. [54]	2012	Amenorrhea	24	CT + triptorelin CT alone	27 22	39 38	No protection
Elgindy et al. [59]	2013	Amenorrhea	12	CT + triptorelin (±GnRHa antagonist) CT alone	50 50	33 32	No protection
Song et al. [55]	2013	Amenorrhea and postmenopausal levels of FSH and E2	12	CT + leuprolide acetate CT alone	89 94	40.3 42.1	Protection
Jiang et al. [56]	2013	Amenorrhea	-	CT + triptorelin CT alone	10 11	- -	Protection
Karimi-Zarchi et al. [57]	2014	Amenorrhea	6	CT + triptorelin CT alone	21 21	37	Protection
Moore et al. [60] Moore et al. [63]	2015 2019	Amenorrhea and postmenopausal levels of FSH	24	CT + goserelin CT alone	105 113	37.6 38.7	Protection
Leonard et al. [61]	2017	Amenorrhea and postmenopausal levels of FSH and E2	Between 12 and 24	CT + goserelin CT alone	103 118	37.9 38.8	Protection
Zhang et al. [62]	2018	Amenorrhea and postmenopausal levels of FSH and E2	36–72	CT + goserelin CT alone	108 108	37.5 39	No protection
Zhong et al. [64]	2019	Amenorrhea	12	CT + goserelin CT alone	51 45	37.0 40.0	Protection

Abbreviations: POI, premature ovarian insufficiency; GnRHa, gonadotropin-releasing hormone agonist; CT, chemotherapy; FSH, follicle-stimulating hormone; E2, estradiol; DFS, disease-free survival; OS, overall survival; OR, odds ratio; CI, confidence intervals; HR, hazard ratio; RR, risk ratio. Except in four trials, all other studies demonstrated a reduction on risk of chemotherapy-induced POI with temporary ovarian suppression with GnRHa during chemotherapy. The largest trials (PROMISE-GIM6 [53,58], POEMS/SWOG S0230 [60,63] and Anglo Celtic Group OPTION [61]) showed consistent results: the addition of GnRHa during chemotherapy provided a 15% absolute reduction in POI rates.

**Table 2:** Available meta-analyses evaluating temporary ovarian suppression with GnRHa during chemotherapy specifically and only in breast cancer patients.

Authors	Year	Disease	No. of Included Studies (no. of RCTs)	No. of Patients	Overall Results
Yang et al. [79]	2013	Breast cancer	5 (5)	528	Protection for POI (not for pregnancy)
Wang et al. [80]	2013	Breast cancer	7 (7)	677	Protection for POI
Vitek et al. [81]	2014	Breast cancer hormone receptor-negative only	4 (4)	252	No protection
Shen et al. [82]	2015	Breast cancer	11 (11)	1062	Protection for POI (not for pregnancy)
Lambertini et al. [83]	2015	Breast cancer	12 (12)	1231	Protection for POI (also for pregnancy)
Munhoz et al. [84]	2016	Breast cancer	7 (7)	856	Protection for POI (also for pregnancy)
Silva et al. [85]	2016	Breast cancer	7 (7) ^a^	1002 ^a^	Protection for POI
Bai et al. [86]	2017	Breast cancer	15 (15) ^a^	1540 ^a^	Protection for POI (also for pregnancy)
Lambertini et al. [87]	2018	Breast cancer	5 (5) ^b^	873	Protection for POI (also for pregnancy)

Abbreviations: GnRHa: gonadotropin-releasing hormone agonist; POI: premature ovarian insufficiency; RCT: randomized controlled trial. ^a^ Data from the original publication (Del Mastro et al. JAMA 2011) and the updated analysis (Lambertini M et al. JAMA 2015) of the PROMISE-GIM6 trial were considered twice instead of as from the same study. ^b^ Based on individual patient-level data.

**Table 3:** Randomized trials evaluating temporary ovarian suppression with GnRHa during chemotherapy in patients with malignancies other than breast cancer.

Authors	Year	Disease	POI Definition (Timing of Its Evaluation)	Timing POI Evaluation (Months)	Treatment Regimen	No. Patients	Median Age (Years)	Overall Results
Waxman et al. [89]	1987	HL	Amenorrhea	Up to 36	CT + buserelin CT alone	8 10	28.5 25.9	No protection
Giuseppe et al. * [75]	2007	HL	Amenorrhea	NR	CT + triptorelin CT alone	14 15	24.3 24.3	No protection
Gilani et al. [90]	2007	Ovarian Cancer	Amenorrhea and postmenopausal levels of FSH	6	CT + triptoreline CT alone	15 15	21 22	Protection
Behringer et al. [76]	2010	HL	AMH levels below normal range	12	CT + goserelin CT + OC	11 12	25.3 26.0	No protection
Demeestere et al. [77] Demeestere et al. [78]	2013 2016	HL and NHL	Amenorrhea	12	CT + triptorelin + OC CT + OC	45 39	25.6 27.3	No protection

Abbreviations: POI, premature ovarian insufficiency; GnRHa, gonadotropin-releasing hormone agonist; HL, Hodgkin lymphoma; NHL, non-Hodgkin lymphoma; AMH, anti-Müllerian hormone; FSH, follicle-stimulating hormone; CT, chemotherapy; OC, oral contraceptives; OS, overall survival; OR, odds ratio; CI, confidence intervals. * The inconsistencies in methods and results pose strong doubts about the randomized nature of the study.

**Table 4:** Available meta-analyses assessing temporary ovarian suppression with GnRHa during chemotherapy in patients with malignancies other than breast cancer or not restricted to only breast cancer trials.

Authors	Year	Disease	No. of Included Studies (no. of RCTs)	No. of Patients	Overall Results
Clowse et. al. [93]	2009	Autoimmune diseases, HL and NHL	9 (2)	366	Protection for POI
Ben-Aharon et al. [94]	2010	Autoimmune diseases, breast cancer, HL and NHL	16 (5)	681	Protection for POI (not in RCTs)
Kim et al. [95]	2010	Autoimmune diseases, breast cancer, HL and NHL	11 (3)	654	Protection for POI
Bedaiwy et al. [96]	2011	Breast cancer, ovarian cancer and HL	6 (6)	340	Protection for POI (not for pregnancy)
Zhang et al. [97]	2013	HL and NHL	7 (3)	434	Protection for POI (not for pregnancy)
Sun et al. [98]	2014	Breast cancer, ovarian cancer and HL	8 (8)	621	Protection for POI (not for pregnancy)
Del Mastro et al. [99]	2014	Breast cancer, ovarian cancer, HL and NHL	9 (9)	765	Protection for POI
Elgindy et al. [59]	2015	Breast cancer, ovarian cancer, HL and NHL	10 (10)	907	No protection
Senra et al. [100]	2018	Breast cancer, HL and NHL	13 (13)	1208	Protection for POI (also for pregnancy)
Hickman et al. [101]	2018	Breast cancer, ovarian cancer, HL and NHL	10 (10)	1051	Protection for POI
Sofiyeva et al. [102]	2019	Autoimmune diseases, breast cancer, HL and NHL	18 (11)	1043	Protection for POI
Zheng et al. [103]	2019	Breast cancer, HL and NHL	12 (12)	1413	Protection for POI (not for pregnancy)
Chen et al. [104]	2019	Breast cancer, ovarian cancer and HL	12 (12)	1369	Protection for POI (not for pregnancy)
Luong et al. [105]	2020	Autoimmune diseases	3 (1)	93	Protection for POI

Abbreviations: POI, premature ovarian insufficiency; GnRHa, gonadotropin-releasing hormone agonist; HL, Hodgkin lymphoma; NHL, non-Hodgkin lymphoma.

**Table 5:** Overview of the main concepts concerning the use of GnRHa in reducing the risk of POI in patients undergoing gonadotoxic treatments.

Questions	Summary
How can we estimate the risk of treatment-induced gonadotoxicity?	The risk of treatment-induced gonadotoxicity is influenced by patient-related (i.e., age and genetic) and treatment-related (i.e., type and dose of chemotherapy) factors. Limited data exist to date on the possible gonadotoxic effect of new drug such as monoclonal antibodies or *PARP* inhibitors.
Is de-escalation of cancer treatment a valid and safe option to reduce treatment-related gonadotoxicity?	Due to the development of increasingly individualized anticancer therapies, particularly for early-stage cancer, a growing attention has been paid in tailoring type and intensity of systemic therapies, balancing between the individual risk of cancer relapse and toxicity. In some setting, de-escalation of chemotherapy is possible with lower rate of treatment-induced amenorrhea in front of similar survival outcomes.
Is ovarian suppression with GnRHa during chemotherapy a valid and safe option to reduce treatment-related gonadotoxicity?	Use of GnRHa during treatment aiming to prevent chemotherapy-induced POI has been evaluated in different diseases with a special focus in breast cancer patients. In this setting, most of the trials demonstrated a reduction in the risk of chemotherapy-induced POI, irrespective of hormone receptor status. This technique has been considerably less investigated in women with malignancies other than breast cancer, with mostly negative results. Overall, based on its efficay and safety data, this technique should be offered to patients that receive chemotherapy and want to preserve ovarian function, particulalry to those with breast cancer.

Abbreviations: GnRHa, gonadotropin-releasing hormone agonist; POI, premature ovarian insufficiency.

## Data Availability

Data sharing not applicable. No new data were created or analyzed in this study. Data sharing is not applicable to this article.
